# Machine learning and logistic regression in estimating survival in patients with high-malignant deep-seated soft tissue sarcomas: development and analysis based on a population-based retrospective cohort

**DOI:** 10.2340/17453674.2026.45509

**Published:** 2026-03-10

**Authors:** Andrea THORN, Jessica A LAVERY, Thomas BAAD-HANSEN, Jonathan A FORSBERG, Michael Mørk PETERSEN, Christina Enciso HOLM

**Affiliations:** 1 Department of Orthopaedic Surgery, Rigshospitalet – University of Copenhagen, Copenhagen, Denmark; 2 Department of Epidemiology and Biostatistics, Memorial Sloan Kettering Cancer Center, New York, USA; 3 Department of Orthopaedic Surgery, Aarhus University Hospital, Aarhus, Denmark; 4 Department of Orthopedic Surgery, Orthopaedic Service, Oncology, Memorial Sloan Kettering Cancer Center, New York, USA

## Abstract

**Background and purpose:**

Soft tissue sarcomas are a heterogeneous group of malignant tumors with a high risk of metastasis, primarily to the lungs, making accurate survival prediction an essential part of long-term planning. No machine learning (ML) survival prediction models have been developed using a modern, population-based dataset from Scandinavia. We aimed to develop and compare ML models with logistic regression in predicting 5-year survival in soft tissue sarcoma patients and identify key predictive variables.

**Methods:**

This retrospective cohort study included patients diagnosed with deep-seated, high-grade soft tissue sarcomas of the extremities and trunk wall in Denmark from 2000 to 2016. Logistic regression was compared with 4 developed ML models, including random forest. Performance was assessed using the area under the curve (AUC), sensitivity, specificity, and calibration metrics, with a 70:30 training–test split and 5-fold cross-validation to evaluate the models.

**Results:**

516 patients were included, of whom 226 (44%) died within 5 years following surgery. Random forest demonstrated the best ML performance on the training set and was compared with logistic regression on the test set. Logistic regression achieved an AUC of 0.74 (95% confidence interval [CI] 0.66–0.82), outperforming random forest‘s AUC of 0.65 (CI 0.56–0.74). Logistic regression also had higher sensitivity (0.65 vs 0.59) and specificity (0.72 vs 0.69), while random forest had a lower Brier score (0.38 vs 0.41).

**Conclusion:**

Although the developed random forest ML model performed well during training, logistic regression outperformed it after internal validation. Soft tissue sarcomas located in the trunk, grade 3 tumors, and chemotherapy within 3 months of surgery demonstrated the highest negative effect on survival, consistent with current treatment protocols in which patients with high-risk disease are managed with more aggressive multimodal therapy. Further external validation and assessment of clinical utility are required before potential clinical implementation.

Sarcomas are a heterogeneous group of malignant tumors originating from connective tissues, with an estimated incidence of 6–8 per 100,000 people, accounting for around 28,000 new cases per year in Europe and about 300 cases annually in Denmark [1,2]. High-grade soft tissue sarcomas typically metastasize to the lungs, the leading cause of sarcoma-specific death [3]. Standard treatment for soft tissue sarcomas of the extremities and trunk wall includes limb-sparing surgery, often combined with radiotherapy. If metastatic disease is present at diagnosis or after surgery, chemotherapy is utilized. Accurate survival prediction tools are essential for guiding clinical decisions and setting expectations for patients and families. Nomograms have traditionally been widely used, but their applicability is limited by population-specific characteristics and are often time-consuming and unavailable in user-friendly formats [4,5]. Machine learning (ML) is a branch of artificial intelligence (AI). It offers promise in developing efficient decision-support tools for sarcoma prognosis, as it can handle large, complex datasets and identify nonlinear patterns [6,7]. Although several models have been proposed and some have been clinically implemented, such as the Sarculator, none have used a modern, population-based dataset of Scandinavian soft tissue sarcoma patients [8-10]. Logistic regression has been the standard approach for predicting survival when complete follow-up is available for all patients, valued for interpretability and robustness [11]. However, ML may outperform logistic regression by handling high-dimensional data and capturing complex interactions.

In the era of personalized medicine, clinicians and patients require reliable, understandable, and clinically applicable predictive models. Comparing model complexity and interpretability is particularly relevant in orthopedic oncology. Predictive models must be externally validated and provide accurate case-by-case estimates rather than broad prognostic groupings such as the TNM staging system. While ML is increasingly applied, many studies lack direct comparison with classic logistic regression models [9,12].

As such, we sought to (i) develop, evaluate, and compare the performance of various ML models with logistic regression in predicting 5-year overall survival in patients with diagnosed soft tissue sarcomas of the extremities and trunk wall using contemporary Danish population-based data, and (ii) investigate which variables contributed most to predicting survival.

## Methods

### Study design and setting

This study is reported following the TRIPOD+AI guidelines (Transparent Reporting of a multivariable prediction model for Individual Prognosis or Diagnosis + artificial intelligence) [13]. This population-based retrospective cohort study utilizes data from the 2 national tertiary referral centers for orthopedic oncology surgery from 2000–2016. In accordance with the Danish social healthcare system all patients diagnosed with sarcoma will be treated at one of the 2 referral centers for orthopedic oncology. We have collected information on patient and tumor characteristics, diagnostic procedures, treatment specifics, such as radiotherapy and chemotherapy, surgical margins, recurrence patterns, and mortality in all patients from the following sources. Since 2009, all soft tissue sarcoma patients in Denmark have been prospectively reported to the Danish Sarcoma Registry (DSR), which is regularly linked to the Danish Civil Registration System (DCR). The DCR is updated daily with information on migration and vital status for all Danish citizens, allowing complete long-term follow-up on emigration and death [14]. Patients treated before 2009 at Aarhus University Hospital were reported prospectively to the Aarhus Sarcoma Registry. This locally based registry was validated in 2013 with a completeness of 99.3% [15]. Although initially collected for clinical and administrative purposes, the data is well suited to this study’s aims, given Denmark’s comprehensive and systematic recording of soft tissue sarcoma cases and complete follow-up. From 2000 to 2009, data from patients treated at Rigshospitalet was retrospectively collected based on identification extracted from the local pathology register. The patient information was matched to the information from the DSR and Aarhus Sarcoma Registry and was collected from patient records, the radiotherapy system ARIA (Varian Medical Systems, Palo Alto, CA, USA), and DCR [14]. ARIA is an oncology information system widely used in radiotherapy departments to document and manage treatment courses, including radiotherapy planning and delivery data. The primary outcome for this study is 5-year overall survival following surgery in patients with high-malignant deep-seated soft tissue sarcomas of the extremities and trunk wall. Given the study’s retrospective nature, blinding in outcome assessment was impossible.

### Patient selection and variables

A consecutive cohort of patients diagnosed with soft tissue sarcomas of the extremities and trunk wall from 2000–2016 in Denmark was identified from DSR, Aarhus Sarcoma Registry, and the local pathology register.

We used the following exclusion criteria to create our study population:

1. Patients who did not undergo surgery.
2. Grade 1 or borderline tumors (Trojani [16]).
3. Superficial tumors defined as those located above the muscle fascia.
4. Patients younger than 18 years at the time of operation.
5. Patients with an intralesional or undefined resection after surgery (Enneking classification [17]).
6. Patients where tumor size could not be identified.
7. Tumors located outside the trunk and extremities.
8. Fewer than 5 years of potential follow-up from surgery.

After applying the exclusion criteria, the final study cohort consisted of only patients diagnosed and operated on for deep-seated, high-grade (Trojani grade 2 + 3) soft tissue sarcomas located in the extremities or trunk wall.

The primary outcome variable was overall survival (OS), measured from the date of surgery to the date of death from any cause, until emigration, or the end of the follow-up period (January 1, 2024). Thanks to the DCR, the exact date of death is known for all Danish patients; therefore, all patients were accounted for to a minimum of 5-year follow-up, except 1 person who was excluded. All patients with high-grade soft tissue sarcomas in Denmark are included in a 5- to 10-year follow-up program after initial treatment to discover local recurrence and metastasis.

The pathology report of the primary resection primarily determined the size. If this information was unavailable, MRI descriptions were used. If these did not include a size description, the primary orthopedic surgeon’s preoperative evaluation was used. All patients’ information was available for this article, and there was no missing data.

### Statistics: model development and evaluation

The dataset was divided into a 70% training set and a 30% test set for validation, stratified by outcome to ensure survival was appropriately represented in both the training and test sets. The division into 70% training and 30% test sets was chosen to ensure a sufficient number of events for internal validation using the test set. The following models were evaluated: (i) Logistic regression; (ii) Random forest [18]; (iii) Gradient-boosted classification tree (Gbm); (iv) Naïve Bayes; and (v) Support Vector Machine (SVM). The models were selected because they are widely used in ML applications for binary outcomes and are frequently reported in survival prediction research within orthopedic oncology [9,12,19]. 9 variables related to demographics, clinicopathologic characteristics, and treatment were chosen from the dataset and included as potential predictors. Demographic variables included age at surgery and sex. Clinicopathological characteristics included tumor size in centimeters, grade (2 or 3, Trojani [16]), tumor location (upper extremities, lower extremities, and trunk), surgical margin (wide, marginal), and histology (histologies with < 30 patients collapsed into an “other” category). These variables were chosen because they are established prognostic factors for survival in soft tissue sarcomas [20,21]. Treatment-related variables were defined as receipt of chemotherapy or radiotherapy initiated within 3 months of primary surgery, reflecting standard postoperative clinical practice in which adjuvant therapy is typically commenced in the early postoperative period following resection in patients with high-grade soft tissue sarcoma [22]. Continuous covariates were center-scaled and standardized as part of data preprocessing to ensure that all predictors were on a comparable scale and to improve model stability [23]. To robustly evaluate the performance of each method and reduce overfitting, 5-fold cross-validation repeated 5 times was applied to the training set [24,25]. This procedure divides the training data into 5 subsets, allowing the model to be trained on 4 sections and validated on the remaining partition of the data. The tuning parameters that achieved the highest area under the receiver operating characteristic curve (AUC) during cross-validation were then used to train the final model on the full training set. The 2 best-performing models were subsequently evaluated on the independent 30% test dataset. Model performance was evaluated using sensitivity, specificity, positive predictive value, negative predictive value, receiver operating characteristic (ROC) analysis, and AUC. An AUC ≥ 0.7 was used as a commonly applied heuristic to contextualize discriminatory performance rather than as a criterion for clinical applicability [24-26]. Calibration and goodness-of-fit were assessed using calibration curves, in which predictions were compared with observed outcomes [27]. Model accuracy was further quantified using the Brier score, which measures the accuracy of survival predictions, with lower scores indicating better performance [28]. Receiver operating curves and calibration curves are only reported on the test data. Detailed descriptions of model development are provided in the Appendix. No prespecified clinical decision thresholds were defined for sensitivity, specificity, positive predictive value, or negative predictive value, as the models were evaluated for comparative performance rather than clinical implementation.

Analyses were performed using the *tidy models* framework [29] R, version 4.2.0 (R Development Core Team, Vienna, Austria, 2020).

### Ethics, registration, data sharing, funding, AI use, and disclosures

The study was conducted according to the Helsinki Declaration and approved by the Danish Data Protection Agency (Videnscenter for Dataanmeldelser) (P-2022-549). Patient consent was waived as it was not required according to Danish law. Support for this work was provided to Memorial Sloan Kettering Cancer Center by a core grant from the National Cancer Institute (P30 CA008748), and “Rigshospitalets Forskningsfond” provided a grant to cover the salary for 1 PhD student (AT). ChatGPT, GPT-4o (OpenAI), and Grammarly (Grammarly In, San Francisco, CA, USA) were used for manuscript language editing and proofreading.

The de-identified dataset used in this study and a detailed data dictionary defining each variable may be available upon reasonable request, subject to specific conditions to protect patient confidentiality. Requests should be directed to the corresponding author and must include a clear research proposal outlining the intended data use. Data is not publicly accessible due to confidentiality restrictions, although the dataset for the 2009–2016 period can be applied for through the Danish Clinical Quality Program (RKKP) at https://www.rkkp.dk/. No patients or members of the public were involved in any aspect of this study, including its design, conduct, reporting, interpretation, or dissemination. This decision was based on the study’s methodological requirements and its focus on retrospective data analysis.

The authors declare no conflict of interest. Complete disclosure of interest forms according to ICMJE are available on the article page, doi: 10.2340/17453674.2026.45509

## Results

### Patients and tumor characteristics

**Figure 1:**
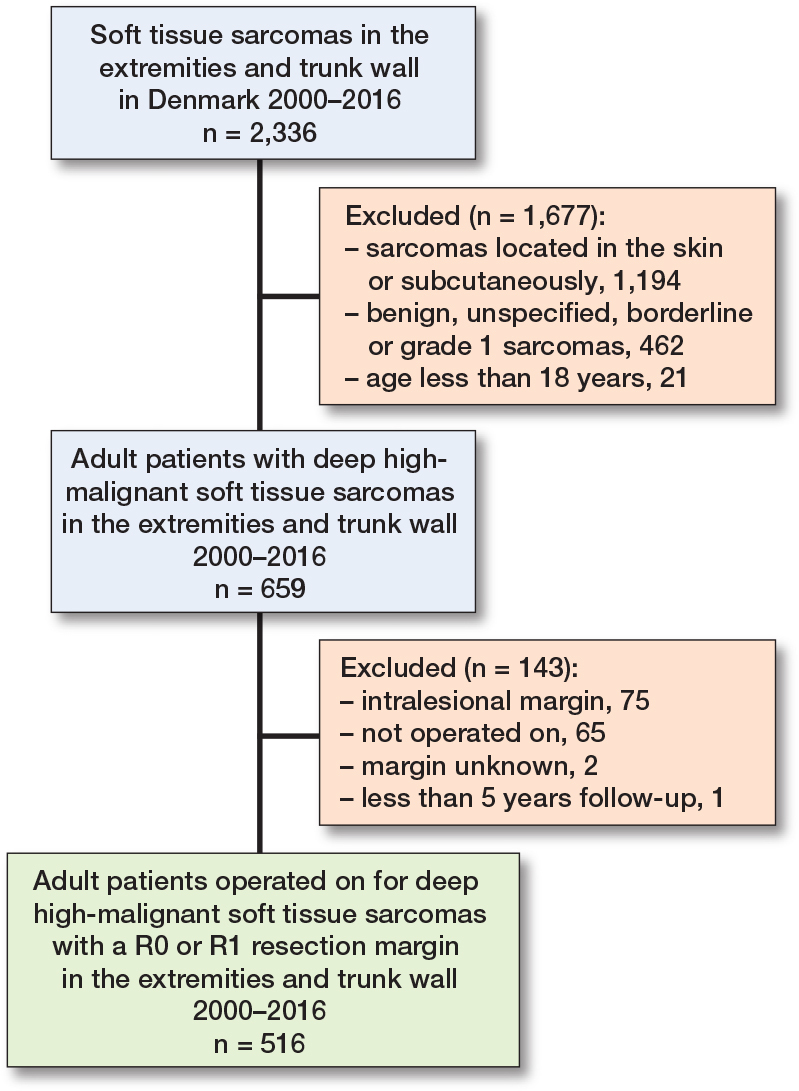
Patient flowchart. R0 resection margin: no cancer cells microscopically at the edge of the removed tissue. R1 resection margin: microscopic examination shows cancer cells at the margin.

The final study cohort consisted of 516 patients diagnosed and operated on for deep-seated, high-grade (Trojani grade 2 + 3) soft tissue sarcomas located in the extremities or trunk wall (Figure 1). Patient demographics data and tumor characteristics for the training and test set are presented in Table 1. 516 patients met the inclusion criteria, and the median age for the overall population was 63 years (interquartile range [IQR] 49–73). 232 (45%) were females; the median tumor size was 9.5 cm (range 1–40; IQR 6.5–13.5); the primary location of the tumor was most frequently the lower extremity 362 (70%), followed by upper extremity 103 (20%) and trunk 51 (10%). The most common histological types were undifferentiated pleomorphic sarcoma (UPS) 206 (40%), synovial sarcoma 50 (10%), myxoid liposarcoma 48 (9.3%), leiomyosarcoma 47 (9.1%), and myxofibrosarcoma 30 (5.8%). Clinical and demographic characteristics were well balanced across the training and test data (Table 1). Overall, 226 (44%) patients were not alive by 5 years post-surgery: there were 158 deaths (44%) among the 361 patients in the training set and 68 (44%) deaths among the 155 patients in the test set.

**Table 1:** Comparison of demographics and clinical variables in the overall patient population and the training and test set. Values are count (%) or as specified

Characteristics	Overall (n = 516)	Training set (n = 361)	Test set (n = 155)
Age at surgery, median (IQR)	63 (49–73)	63 (50–74)	61 (49–71)
Female sex	232 (45)	156 (43)	76 (49)
Size (cm), median (IQR)	9.5 (6.5–13.5)	9 (6–13)	10 (6.5–14)
Histological grade			
Grade 2	157 (30)	120 (33)	37 (24)
Grade 3	359 (70)	241 (67)	118 (76)
Location			
Lower extremity	362 (70)	252 (70)	110 (71)
Truncal	51 (9.9)	33 (9.1)	18 (12)
Upper extremity	103 (20)	76 (21)	27 (17)
Surgical margin			
Wide	260 (50)	186 (52)	74 (48)
Marginal	256 (50)	175 (48)	81 (52)
Chemotherapy within			
3 months of surgery	30 (5.8)	17 (4.7)	13 (8.4)
Radiotherapy within			
3 months of surgery	299 (58)	204 (57)	95 (61)
Histological diagnosis			
UPS	206 (26)	144 (40)	62 (26)
Synovial sarcoma	50 (10)	32 (8.9)	18 (12)
Myxoid liposarcoma	48 (9.3)	36 (10)	12 (7.7)
Leiomyosarcoma	47 (9.1)	35 (9.7)	12 (7.7)
Myxofibrosarcoma	30 (5.8)	20 (5.5)	10 (6.5)
Other	135 (26)	94 (26)	41 (26)

UPS: Undifferentiated pleomorphic sarcoma

### Model performance and selection

#### Training set performance

Logistic regression demonstrated an AUC of 0.77 (95% confidence interval [CI] 0.72–0.82), an NPV of 0.71 (CI 0.65– 0.77), PPV of 0.66 (CI 0.58–0.74), a sensitivity of 0.61 (CI 0.53– 0.68), and a specificity of 0.76 (CI 0.69–0.82). The Brier score was 0.42 (CI 0.40–0.45), reflecting moderate calibration. These results indicate balanced performance across discrimination and calibration metrics for the logistic regression model.

Among the machine learning models, random forest exhibited the highest AUC of 0.94 (CI 0.91–0.96). It also achieved an NPV of 0.89 (CI 0.84–0.93), PPV of 0.87 (CI 0.81–0.92), sensitivity of 0.85 (CI 0.79–0.91), and specificity of 0.90 (CI 0.85–0.94). However, its Brier score of 0.57 (CI 0.54–0.59) indicates potential overfitting, which may affect its calibration. The Gradient–Boosted Classification Tree (GBM) had the lowest Brier score of 0.37 (CI 0.35–0.38). It achieved an AUC of 0.92 (CI 0.89–0.94), comparable to random forest, alongside a high specificity of 0.93 (CI 0.89–0.96) and PPV of 0.88 (CI 0.81–0.93). However, its lower sensitivity of 0.65 (CI 0.57–0.72) and NPV of 0.77 (CI 0.71–0.82) suggest a reduced ability to detect all high-risk patients (Table 2).

**Table 2:** Model performance in the training set. Values are mean (95% confidence interval)

Metric	Logistic regression	Random fores	Gradient-boosted classification tree	Naïve Bayes	Support Vector Machine
AUC	0.77 (0.72–0.82)	0.94 (0.91–0.96)	0.92 (0.89–0.94)	0.80 (0.75–0.84)	0.77 (0.72–0.82)
Brier score	0.42 (0.40–0.45)	0.57 (0.54–0.59)	0.37 (0.35–0.38)	0.48 (0.45–0.51)	0.38 (0.36–0.40)
Negative predictive value	0.71 (0.65–0.77)	0.89 (0.84–0.93)	0.77 (0.71–0.82)	0.72 (0.66–0.77)	0.71 (0.65–0.77)
Positive predictive value	0.66 (0.58–0.74)	0.87 (0.81–0.92)	0.88 (0.81–0.93)	0.74 (0.65–0.81)	0.69 (0.60–0.77)
Sensitivity	0.61 (0.53–0.68)	0.85 (0.79–0.91)	0.65 (0.57–0.72)	0.58 (0.49–0.65)	0.59 (0.51– 0.67)
Specificity	0.76 (0.69–0.82)	0.90 (0.85–0.94)	0.93 (0.89–0.96)	0.84 (0.78–0.89)	0.79 (0.73– 0.85)

AUC: area under the receiver operating characteristic curve.

**Figure 2:**
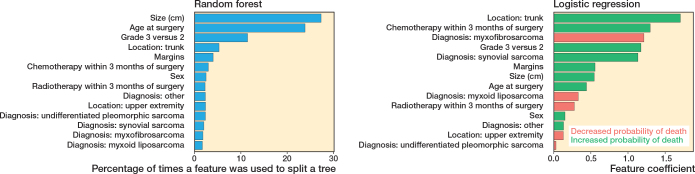
Coefficients of machine learning (random forest) and logistic regression. The random forest bar plot displays feature importance based on the frequency with which each feature was used to split nodes in the decision trees. In the logistic regression bar plot, positive coefficients (green) indicate increased probability of death, while negative coefficients (red) indicate decreased probability of death.

After evaluating the training set, the random forest model was selected for further assessment, along with the logistic regression model, on the test set. Variable importance is reported for each model (Figure 2). For logistic regression, the location of a tumor in the trunk has the highest positive coefficient, suggesting a significantly increased risk of death. Unlike logistic regression, feature importance in random forests does not convey direction (increasing or decreasing risk) but highlights the relevance of predictors in decision-making. It demonstrates that the tumor size was the most important feature in this model.

#### Test set performance

**Figure 3:**
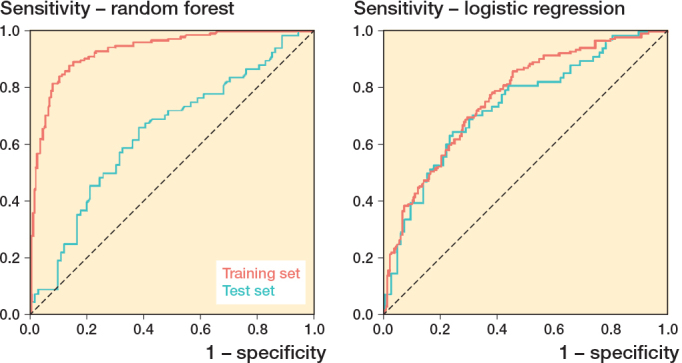
Receiver operating characteristic (ROC) curves for machine learning random forest models and logistic regression in the training and test sets.

**Figure 4:**
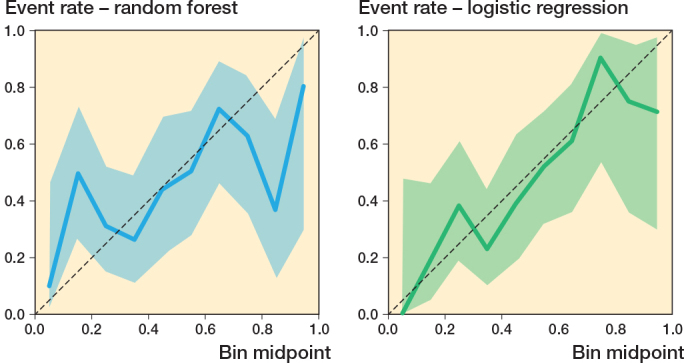
Calibration plots for machine learning (random forest) and logistic regression on the test data.

The logistic regression model achieved an AUC value of 0.74 (CI 0.66–0.82) (Figure 3). Sensitivity and specificity were 0.65 (CI 0.52–0.76) and 0.72 (CI 0.62–0.81). PPV was 0.65 (CI 0.52–0.76), and NPV was 0.72 (CI 0.62–0.81) (Table 3). The Brier score for this model was 0.41 (CI 0.37–0.45), and the calibration plot showed alignment with the reference line in mid-range probabilities (0.3–0.7) (Figure 4). The random forest model demonstrated an AUC value of 0.65 (CI 0.56–0.74) (Figure 3). Sensitivity was 0.59 (CI 0.46–0.71), and specificity was 0.69 (CI 0.58–0.78). The model’s PPV was 0.60 (CI 0.47–0.72), and NPV was 0.68 (CI 0.57–0.78) (Table 3). The random forest model had a Brier score of 0.38 (CI 0.34–0.42), and its calibration plot closely followed the reference line across most probability ranges (Figure 4).

**Table 3:** Model performance in the test set on the selected models from the training set. Values are mean (95% confidence interval)

Metric	Logistic regression	Random forest
AUC	0.74 (0.66–0.82)	0.65 (0.56–0.74)
Brier score	0.41 (0.37–0.45)	0.38 (0.34–0.42)
Negative predictive value	0.72 (0.62–0.81)	0.68 (0.57–0.78)
Positive predictive value	0.65 (0.52–0.76)	0.60 (0.47–0.72)
Sensitivity	0.65 (0.52–0.76)	0.59 (0.46–0.71)
Specificity	0.72 (0.62–0.81)	0.69 (0.58–0.78)

AUC: see Table 2

## Discussion

After internal validation, logistic regression outperformed the random forest model across discrimination and threshold-specific diagnostic measures. Although confidence intervals overlapped, logistic regression demonstrated higher AUC, sensitivity, specificity, PPV, and NPV on the test set, indicating more reliable discriminatory power and classification of survivors and non-survivors. Calibration was acceptable for both models, with the random forest demonstrating a slightly lower Brier score; however, this did not compensate for the demonstrated inferior discriminatory and diagnostic performance. Our findings suggest that the increased complexity of the random forest model did not translate into improved predictive benefit, and that the classic logistic regression model provided more robust and clinically reliable performance in the present dataset.

The logistic regression ranked tumors in the trunk, patients receiving chemo within 3 months of surgery, and grade 3 tumors as having the highest negative effect on survival (see Figure 1). The random forest model outputs feature importance based on how frequently each variable is used to split decision trees. Size (cm) was most often used in tree splits and had the most important impact on survival predictions (see Figure 1). These predictors align with the other studies on soft tissue sarcoma survival, indicating that both models’ results align with the literature [21,30].

Many studies on ML for survival prediction in soft tissue sarcoma patients do not reference NPP, NVP, sensitivity, and specificity, thereby not giving the reader all the information on discrimination needed to make calculated decisions on what model to use [8,9,12]. We believe that multiple performance metrics provides a more complete assessment of model performance, as each metric compensates for the limitations of the others. Specifically, the AUC summarizes overall discrimination across all possible thresholds but does not reflect model performance at a selected clinical decision threshold or provide information on predictive values. Furthermore, sensitivity and specificity quantify the trade-off between false negatives and false positives at a chosen threshold, independent of disease prevalence. In contrast, PPV and NPV incorporate the prevalence of the outcome and therefore reflect the clinical applicability of the model in terms of the probability that a prediction is correct. However, PPV and NPV do not directly describe the balance between missed outcomes and overestimation of risk. As such, the metrics do not necessarily move in parallel; a model may show high sensitivity and specificity yet poor predictive value depending on the prevalence. The importance of multiple performance measures is highlighted in the present study: although the AUC for logistic regression was 0.74, this alone does not guarantee optimal clinical applicability or predictive reliability. By evaluating threshold-specific measures, the superior classification performance and clinical reliability of the logistic regression model is demonstrated.

Standards of practice for the design of machine learning models are warranted, including sample size. Large sample sizes have previously been recognized as the most influential factor [31,32]. Studies comparing traditional statistical models with ML models suggest that regression models can outperform ML methods in survival prediction when small to medium sample sizes and the number of predictors are limited [19,33]. In contrast, ML techniques are often considered advantageous in large, heterogeneous datasets, such as clinical registries, where complex non-linear relationships and missing data are common [34,35].

Random forest models can handle incomplete data, which is common in the clinical setting, and have been the model of choice for many predictive models [36]. In addition, machine learning models can automatically explore non-linear effects and interaction patterns among predictors, making them more flexible than traditional regression approaches, in which relationships between covariates and outcomes must be explicitly specified.

This flexibility, however, comes at the cost of an increased risk of overfitting, particularly in smaller datasets. Consequently, large sample sizes are often required to ensure model stability and generalizability during model training [37,38]. In the present study the dataset was complete, with no missing data, and included all nationally registered patients with high-grade, deep-seated soft tissue sarcomas treated between 2000 and 2016. As a result, the ability of ML models to handle missing or irregular data did not confer a clear advantage in this context.

### Limitations

Although the cohort included tumors with heterogeneous histological subtypes, many subgroups were represented by relatively small numbers of patients, which may further limit the ability of more complex models to reliably capture higher-order interaction effects [39]. To mitigate this, we ensured that the training and test sets were balanced with respect to baseline and treatment characteristics, applied 5-fold cross-validation to reduce overfitting, and restricted the cohort to patients treated according to standardized protocols with uniform follow-up. No patients were lost to follow-up. All patients in the dataset were treated following the same standardized treatment protocol and underwent uniform follow-up, reducing irregularity in treatment and monitoring outcomes regardless of histological subtype.

This study was not designed to establish clinical decision thresholds or to support direct clinical implementation of a prognostic model. Rather, it provides a comparative evaluation of commonly used statistical and machine-learning approaches for survival prediction in a well-defined, population-based orthopedic oncology cohort. Formal assessment of clinical utility, including decision-analytic evaluation and external validation, will be required prior to any potential clinical use. Although this is a retrospective study, the risk of subjective bias is eliminated due to the high completeness and regular validation through the systematic recording in medical registries, medical journals, and databases in Denmark. Ensuring the inclusion of all national sarcoma patients, regardless of setting, makes the data highly representative of the Danish soft tissue sarcomas population. Furthermore, Denmark has a centralized and universal healthcare system, which minimizes selection bias due to private care or fragmented treatment pathways. The representativeness of the data is, therefore, strong and suitable for generalization to similar population-based healthcare settings. However, care should be taken when applying the results to countries with more fragmented healthcare systems.

### Conclusions

Logistic regression demonstrated superior validity and discrimination compared with machine-learning techniques in the present 5-year survival models, likely reflecting the robustness of logistic regression in smaller, well-curated datasets that meet its underlying assumptions. Soft tissue sarcomas in the trunk, chemotherapy within 3 months of surgery, and grade 3 tumors demonstrated the highest negative effect on survival.

*In perspective*, we believe that prediction models such as these may serve as valuable clinical decision-support tools by providing clinicians and patients with information relevant to the management of soft tissue sarcomas. In Denmark, follow-up programs currently differentiate patients based solely on tumor grade (grade 1 vs grade 2–3), with the exception of myxoid liposarcoma, which is allocated longer follow-up including retroperitoneal imaging. Developing predictive models informed by the variable importance identified in this study may support future efforts to refine risk stratification beyond tumor grade alone. Such approaches could allow consideration of additional factors, including tumor location, size, oncological treatment, and patient age, when designing follow-up strategies. Although this was not evaluated in the present study, this framework may facilitate more individualized follow-up intensity based on estimated risk, provided that models are externally validated and their clinical utility is formally assessed. We therefore encourage other institutions to perform external validation in non-Scandinavian populations.

## Notes

AT: Formulation/identification of the scientific problem. Development of the key methods. Planning of the experiments and methodology design and development. Conducting the experimental work/clinical studies/data collection/obtaining access to data. Interpretation of the results. Writing of the first draft of the manuscript. Finalisation of the manuscript and submission. CH: Formulation/identification of the scientific problem. Development of the key methods. Interpretation of the results. Finalisation of the manuscript and submission. JF: Planning of the experiments and methodology design and development. Finalisation of the manuscript and submission. JL: Planning of the experiments and methodology design and development. Conducting the analysis of dat. Interpretation of the results. Finalisation of the manuscript and submission. MP: Formulation/identification of the scientific problem. Formulation/identification of the scientific problem. Finalisation of the manuscript and submission. TBH: Conducting the experimental work/clinical studies/data collection/obtaining access to data. Finalisation of the manuscript and submission

Handling co-editors: Paul Gerdhem and Philippe Wagner

*Acta* thanks Alejandro Ignacio Marcano and other anonymous reviewers for help with peer review of this manuscript.

## Appendix

### Hyperparameter tuning

To enhance the robustness of the models, 5-fold cross-validation was repeated 5 times on the training dataset. Division of the data into folds was stratified by the outcome in order to ensure that survival outcomes were proportionally represented across folds. After 5-fold cross-validation, the random forest model was fit with the following hyperparameters: fraction (mtry): 6, trees: 1,373, and min n 13.

### Model performance assessment

Model discrimination was assessed based on sensitivity, specificity, positive predictive value, negative predictive value, and receiver operating characteristic (ROC) analysis, including area under the curve (AUC). Discrimination measures with ROC analysis and AUC measure how well the model can separate those likely to experience the outcome, in our case death, from those not experiencing it [26]. The value ranges from 0–1, where 1–0.8 is perfect discrimination, 0.8–0.7 is acceptable, and 0.7–0.5 is poor. A value of 0.5 equals a 50% chance of discrimination, making it no better than chance, and values < 0.5 indicate that the model is performing worse than random chance [40]. Models are often considered suitable for clinical usage if the AUC ≥ 0.7 [24,25].

Model accuracy was reported using the Brier score, which summarizes overall performance in prediction models by quantifying the accuracy of the predicted probability of death by 5 years vs the observed survival status at 5 years. The reported values are between 0 and 1, with 0 indicating that the model is perfectly accurate in predicting deaths at 5 years and 1 indicating that the model is perfectly inaccurate in predicting deaths at 5 years. A score of 0.25 reflects a predicted 50% incidence of outcome and is considered non-informative [28]. The goodness-of-fit or calibration was performed on all models selected for the test set. This assesses the agreement between the model’s predicted risk and the observed outcomes in the patient data. Calibration curves are presented, in which the x-axis represents the predicted risk and the y-axis the observed risk. The closer the prediction is to the 45° diagonal line, the better the model is calibrated [27].
